# Genomic Prediction of Seed Quality Traits Using Advanced Barley Breeding Lines

**DOI:** 10.1371/journal.pone.0164494

**Published:** 2016-10-26

**Authors:** Nanna Hellum Nielsen, Ahmed Jahoor, Jens Due Jensen, Jihad Orabi, Fabio Cericola, Vahid Edriss, Just Jensen

**Affiliations:** 1 Nordic Seed A/S, Grindsnabevej 25, 8300, Odder, Denmark; 2 Department of Molecular Biology and Genetics—Center for Quantitative Genetics and Genomics, Aarhus University, Blichers Allé 20, 8830, Tjele, Denmark; 3 Department of Plant Breeding, The Swedish University of Agricultural Sciences, 2353, Alnarp, Sweden; Institute of Genetics and Developmental Biology Chinese Academy of Sciences, CHINA

## Abstract

Genomic selection was recently introduced in plant breeding. The objective of this study was to develop genomic prediction for important seed quality parameters in spring barley. The aim was to predict breeding values without expensive phenotyping of large sets of lines. A total number of 309 advanced spring barley lines tested at two locations each with three replicates were phenotyped and each line was genotyped by Illumina iSelect 9Kbarley chip. The population originated from two different breeding sets, which were phenotyped in two different years. Phenotypic measurements considered were: seed size, protein content, protein yield, test weight and ergosterol content. A leave-one-out cross-validation strategy revealed high prediction accuracies ranging between 0.40 and 0.83. Prediction across breeding sets resulted in reduced accuracies compared to the leave-one-out strategy. Furthermore, predicting across full and half-sib-families resulted in reduced prediction accuracies. Additionally, predictions were performed using reduced marker sets and reduced training population sets. In conclusion, using less than 200 lines in the training set can result in low prediction accuracy, and the accuracy will then be highly dependent on the family structure of the selected training set. However, the results also indicate that relatively small training sets (200 lines) are sufficient for genomic prediction in commercial barley breeding. In addition, our results indicate a minimum marker set of 1,000 to decrease the risk of low prediction accuracy for some traits or some families.

## Introduction

Barley (*Hordeum vulgare*) is one of the major cereals and it is used for multiple purposes such as feed, food and malting [1,2]. In the northern part of Europe, having high malting quality is extremely important for a spring barley variety. Therefore, both quality and yield improvement are breeding goals in barley breeding programs in Northern Europe. Both are complex traits controlled by multiple quantitative trait loci (QTL) most of them with a small effect on the phenotype [3,4]. QTL mapping studies for yield and quality parameters in barley detected multiple QTL responsible for malting quality parameters and yield parameters such as grain yield, test weight (TW) and seed size fractions [5]. One limitation of QTL mapping and marker assisted selection (MAS) is that the QTL identified have relatively small effects and therefore explain a small part of the genetic variation [6,7]. In addition, a limited set of markers cannot cover all parts of the genome; therefore the amount of genetic variance explained by markers is often limited. A supplementary or alternative method is genomic selection which has been of growing interest (one example is [8]). Genomic selection was first developed in animal breeding [9], and is now widely used in many animal breeding programs. The authors of [10] were some of the first to show the benefit of genomic selection in plants. They completed a simulation study on maize, where they indicated a benefit of using genomic selection instead of traditional marker assisted selection [10]. Since then, a number of studies investigated the use of genomic selection in wheat [11–13]. However, only a few studies have investigated genomic selection in barley (examples are [14,15])

Genomic prediction has the benefit of using information based on the total marker information without need of significant single markers associations. The marker information is used in models to calculate genomic estimated breeding values (GEBVs) which can assist the breeders in their selection decisions [9]. The GEBVs are calculated as a sum of all markers effects. The procedure is first to train the model by using a training set that both has phenotypic measurements and genotype information available. Secondly, the trained model is applied on the candidates or validation population (VP), where only the genomic information is available. In the process of developing the models a VP is defined by selecting subsets of the available varieties and ignoring the phenotypic records. The correlation between GEBVs and phenotypic records corrected for environmental effects is used to quantify the predictive ability of the model.

One study [16] highlights the accuracy of unknown genotypes as a function of the training set size (N), trait heritability on an entry-mean basis (h^2^), and the effective number of QTL (M_e_).

$$r_{g\;\hat{g}}=\sqrt{\frac{Nh^{2}}{Nh^{2}+M_{e}}}$$ (1)

According to this theory, the accuracy increases with higher heritability. Studies also showed that most traits with high heritability are predicted well [17]. The study [17] estimated the correlation between GEBVs and phenotypic values for several traits in an empirical bi-parental barley population, and found accuracy to increase with increasing trait heritabilities.

Another important parameter influencing the accuracy is the number of individulals included in the training population as indicated by Eq 1. Increased accuracy as a result of a larger training set, has been found to apply for several species [18]. However, the study [18], found that the population structure of the studied species plus the composition of the training set is essential. Hence, family structure and relateness has a high influence on prediction accuracy. In general, high reliabilities are expected when the validation set compose many small families as opposed to a few large ones [19]. Furthermore, a high relationship between training set and validation set will result in higher accuracies [19].

Genetic gains are obtained by selection in the breeding material. Therfore, it is of great interest to predict a new breeding set from previous ones. Consequently, it is relevant to enable prediction in new progeny sets across years and predict across less related breeding sets. However, this issue is a major challenge in genomic prediction in plants, since prediction accuracy between genetically unrelated populations (or families) is often low [20,21]. Investigation of genomic prediction accurracy between families is done by cross-validation strategies, where less related groups are predicted. The authors of [21] performed cross-validation using three different wheat sets originated by different parents: two DH sets and one recombinant inbred set, and found low prediction accuracies when one set was used to predict another.

The traits: seed size fractions, test weight, protein content and protein yield are all important and routinely measured in commercial breeding programs. Seed size and shape is known to be associated with malting performance [22]. One study detected QTL for kernel size and shape in barley on all seven chromosomes [23]. Since plenty of QTL were detected for kernel size and some with slight effects, genomic selection can potentially be beneficial for prediction in seed size traits. Several QTL have also been found for test weight and kernel protein content [5,24], and genomic prediction may also be beneficial for these traits. Few studies have investigated the applicability of genomic selection in barley breeding (one is [25]). This study aims to evaluate the use of genomic prediction models in a commercial barley breeding program. Our main goals were: 1) Investigating prediction accuracy within and across progeny sets. 2) Investigating the prediction accuracy between less related families. 3) Investigating the effects of training population size and number of markers on prediction accuracy.

## Materials and Methods

### Plant material and field trials

The Danish breeding company, Nordic Seed A/S, delivered genetic material and conducted all field trials. The germplasm consisted of 309 advanced spring malting barley lines from Nordic Seed. A total of 119 of the 309 lines were harvested in year 2014, while the remaining (190) were harvested in 2015. The lines in the two harvest years originated from two different sets of parents, and are referred to as S_2014_ and S_2015_. However, the two sets of crossing parents were overlapping; hence, 16 of the parents were used in both years. All lines were grown in two locations in Denmark: Dyngby (Jutland) and Holeby (Lolland). Dyngby is characterized as fine sandy loam (JB6) soil, while Holeby is characterized as loam soil (JB7). The fields were divided in trials consisting of smaller plots, and each trial was a randomized block-design with three replicates of each line. In few cases, phenotypic measurements were only obtained from two of the three replicates. Moreover, two check lines were sown in each of the replications in each trial; thus, there were more replications of these two check lines. Each plot measured 5.5 × 1.5 m (8.25 m^2^) and each plot was harvested separately and a random sample of the seeds harvested were retained for further analysis. The yield was obtained from each plot by total weight of the harvested seeds.

### Phenotypes

Phenotyping was done on the seeds obtained from each plot. The phenotypic measurements included seed size fractions, protein content, protein yield, test weight and ergosterol content. The seed size fractions were obtained using a SORTIMAT (Baumann Saatzuchtbedarf) instrument to separate the seed samples of 100 g into four seed size classes of >2.8, 2.8–2.5, 2.50–2.2 and < 2.2 mm. Seed fractions in each sample were weighted. The smallest fraction (<2.2 mm) was considered as dust and not used in any of the analysis. Test weight, ergosterol content and protein content were all measured using an instrument based on NIT (Near Infrared Transmission)-technology (Grain Analyser for grain and flour: Infratec 1241 (FOSS)). Ergosterol content is based on a test calibration, and is not a verified method. An overview of the phenotypes is given in Table 1.

**Table 1:** Overview of all phenotypes. Based on a population of 309 F6 lines.

Abbreviation	Phenotype explanation	Method
f2.8	Weight (g) of seeds with the size > 2.8 mm	Laboratory screening machine: SORTIMAT (Baumann Saatzuchtbedarf)
f2.5	Weight (g) of seeds with the size > 2.5 & <2.8 mm	
f2.2	Weight (g) of seeds with the size > 2.2 & <2.5 mm	
SSW	Standardized seed weight	
Protein	Protein (TS %)	Grain Analyser for grain and flour: Infratec 1241 (FOSS)
TW	Test weight (kg **·** hl^-1^)	
Ergosterol	Ergosterol content (%)	
PY	Protein yield (hkg **·** ha^-1^)	$\frac{protein\;content}{yield\;(hkg∙{ha}^{-1})}$

The statistical models assume traits to follow a normal distribution. Since the three seed size fraction weights were not following a normal distribution, a standardized seed measure was created. A normally distributed seed size measure (SSW) was calculated based on the inverse cumulative normal distribution function. This was done for each fraction and the two measures of seed size were averaged. Since the predictions were similar between all seed size fractions only the results from SSW are shown.

### Genotypes

Genomic DNA was extracted from each of the 309 lines. Three, two-weeks old seedlings of each line, were bulked and DNA was extracted by a modified CTAB procedure (Cetyl Trimetheyl Ammonium Bromide [26]). All lines were genotyped by the Illumina iSelect 9K barley chip. The genotyping was outsourced to TraitGenetics [27]. A total of 7,864 polymorphic markers were obtained from the 9K barley chip of the 309 barley lines. After editing for minor allele frequency ≥ 5% and missing markers per line > 2 percentage the marker set for analysis included 3,540 markers. A total of 2,836 of the 3,540 markers were mapped, while the rest (704) were unmapped.

### Statistical methods and models

The genomic relationship matrix used in the genomic prediction model was calculated using principles described by [28], as follows. **M** is the matrix that specifies the marker alleles, with the elements of −1, 0, and 1 for the homozygote, heterozygote and opposite homozygote, respectively. **P** (matrix of mean allele frequencies) is calculated by using the frequency of the second allele at locus i (p_i_). I.e. The i’th columns is computed as: *P*_i_ = *j*2(*p*_i_-0.5), where *j* is a vector of ones. **Z** is calculated as **M**-**P**, and mean value of the alleles effects are 0. The final scaled **G**-matrix was calculated as:

$$G=\frac{ZZ\prime }{2\sum p_{i}(1-p_{i})}$$ (2)

A PCA was performed based on the **G**-matrix using the build in R-packages prcomp and a heat map was constructed using the R-package hclust [29].

Linear mixed models with different fixed and random effects were tested by evaluation of maximum likelihood and variance components. The final model used for all phenotypes, except protein yield, was:

$$y=Xt+Z_{1}g+Z_{1}l+Z_{2}c+e$$ (3)

For protein yield the model was:

$$y=Xt+Z_{1}g+Z_{2}c+e$$ (4)

where *y* is the vector of phenotypic observations, *t* is the vector of fixed effects. For SSW, protein and PY the fixed effect was year×location, while for TW and ergosterol it was year×location×trial. **Z**_1_ is the design matrices for random factors, genotypes and lines, *g* is the vector of breeding values $g∼N(0,G\sigma_{g}^{2})$, where **G** is the **G**-matrix; *l* is the vector of lines $l∼N(0,I\sigma_{l}^{2})$, where **I** is the identity-matrix; **Z**_2_ is the design matrix for the G×E, *c* is the vector of G×E interactions, $c∼N(0,I\sigma_{c}^{2})$ and *e* is the vector of random residuals. The residuals were assumed independent normally distributed values described as $e∼N(0,I\sigma_{e}^{2})$.

Variance components due to random effects were estimated by REML. These variances were used to calculate the heritability for the traits. All analysis of mixed linear models were conducted using the DMU Package for Analyzing Multivariate Mixed Model developed by Aarhus University, Center for Quantitative Genetics and Genomics [30,31].

Heritability estimates for the two models (5 and 6) were calculated as:

$$h^{2}=\frac{\hat{\sigma_{g}^{2}}}{\hat{\sigma_{g}^{2}}+\hat{\sigma_{l}^{2}}+\frac{\hat{\sigma_{c}^{2}}}{2}+\frac{\hat{\sigma_{e}^{2}}}{6}}$$ (5)

and

$$h^{2}=\frac{\hat{\sigma_{g}^{2}}}{\hat{\sigma_{g}^{2}}+\frac{\hat{\sigma_{c}^{2}}}{2}+\frac{\hat{\sigma_{e}^{2}}}{6}}$$ (6)

where $\hat{\sigma_{g}^{2}}$ is the genomic additive genetic variance, $\hat{\sigma_{l}^{2}}$ is genetic variance that could not be attributed to the markers, $\hat{\sigma_{c}^{2}}$ is the additive genotype × environment variance, $\hat{\sigma_{e}^{2}}$ is the variance of residuals. The number of environments used for testing varieties was two and six replicates were assumed for each line. I.e. this is the heritability of the line means.

The standard error of heritability estimates was calculated from the inverse of the information matrix related to the model using a Taylor series [32,33]. Maximum prediction accuracy was calculated as the correlation coefficient h, the square root of heritability, which is equal to the expected correlation between line means and true genomic breeding values. The accuracies of the genomic predictions (r_GP_) were calculated as the correlation between the phenotypic values corrected for the fixed effects and the GEBVs when own line performance were not included in the model. The bias was calculated as the regression between the phenotypic values corrected for the fixed effects and GEBVs. A deviation from the expected regression of 1.0 shows if the model yields GEBVs with too large or too small variance.

### Predictive ability and cross-validation

The general ability of predicting the phenotypes of future lines based on their genotypes was tested. For this purpose, a leave-one-out (LOO) cross-validation strategy was used. In the LOO strategy, one individual line is excluded from the training population, and the GEBV of same line is predicted from a model trained on all other lines. This was done for all 309 lines, and the accuracy of the 309 predictions was calculated as the correlation between GEBVs and average phenotype corrected for fixed effects in the model.

The first aim was to test the ability to predict across breeding sets, and was evaluated by using leave-set-out (LSO). In the LSO strategy, individuals from one year were excluded and the remaining individuals from the other year were used as training population. Genomic estimated breeding values were calculated for the individuals in the excluded year. The number of individuals differed between years, with 119 in year 2014 and 190 in year 2015. Results were compared to ten repeated predictions calculated using a leave-set-out (LSO_random_) strategy using random sets of same sizes as the breeding sets, S_2014_ and S_2015_. In the LSO_random_, the standard error of accuracy was calculated as the standard deviation between the ten repeated cross validations.

Secondly, the ability to predict across less related lines was tested by using a strategy of where one family (LFO) was excluded from the training set and predicted. One family was defined as lines having at least one parent in common. A number of 104 parents were present in the set. In total, prediction accuracies and biases could be obtained from 104 families of different sizes. The accuracy and bias is given as the correlation between the GEBVs and the corrected phenotype of all 309 lines.

Thirdly, the effect of both training population size and the size of the marker set on prediction accuracy was studied. For this purpose, a LOO cross-validation strategy was used. The minimum size of the training population was found by performing predictions using randomly selected reduced populations. The starting population was the full set of 309 lines that was then reduced nine times by 10% each time. For each reduction step the prediction ability was obtained 100 times using 100 different randomly selected lines. The minimum marker number was investigated using the same principle. The starting set included 3,540 markers, and predictions were made by using reduced marker sets. The size of the marker set was reduced by 10% for each reduction step, and the prediction accuracy was obtained 100 times for each reduction step, using 100 different randomly selected marker sets. The markers were selected such that they were equally distributed on all seven chromosomes, and so that there was at least one cM between selected markers. For the larger marker sets, the minimum distance was reduced, when required. Since variance estimates could not be obtained by using small datasets, variance components and heritability were estimated using the full marker set of 3,540 markers, and predictions were performed by using reduced marker sets and training population.

## Results

### Genotypes

**Fig 1:**
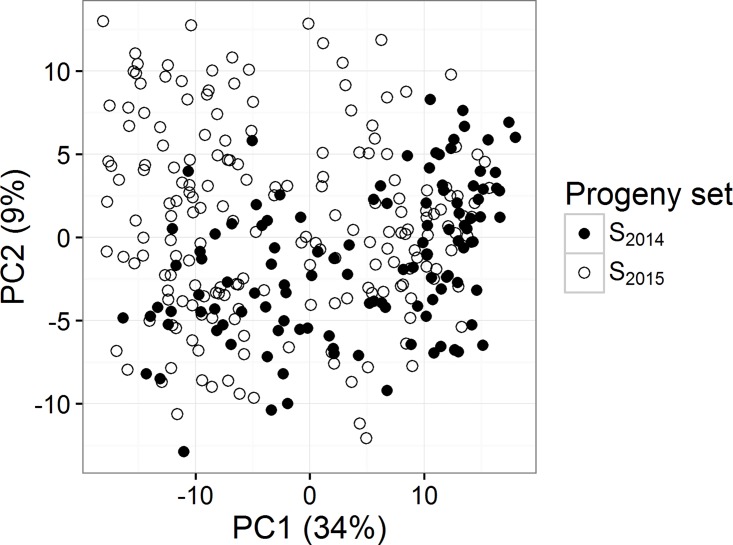
PCA. Relation between the 309 barley lines based on 3,540 SNP markers.

**Fig 2:**
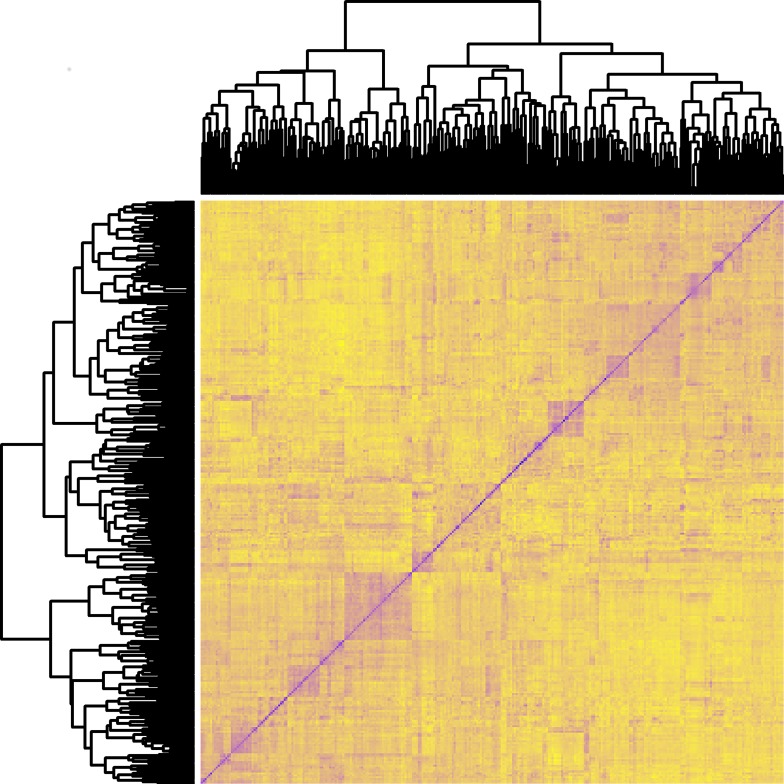
Heatmap. Relationship between the 309 barely lines based on 3,540 SNP markers.

To gain information about the genetic relationship among lines, a PCA was made based on the 3,540 markers (Fig 1). No clear groups are to be seen from the PCA, but the first two principal components explain 34% and 9% of the total variance indicating considerable relationships among lines. The 2014 and 2015 lines were not separated by the PCA-plot, which indicates the absence of a clear family substructure in the data. To confirm the missing genetic grouping, a heat map of the 309 lines is seen as Fig 2. The heat map does not reveal any large genetically different subgroups in the population. Hence, most lines are genetically related to several other lines.

### Phenotypes

The phenotypes, seed size fractions, test weight, ergosterol content, protein content and protein yield were obtained from 309 lines over two years. In each year, three replicates were harvested in two different locations. A summary of the phenotypic values is given in Table 2.

**Table 2:** Summary of phenotypic data. Mean ± standard deviation (SD) of the traits are given as a mean of 2,158 samples representing 309 lines in three replicates on two locations (Dyngby and Holeby) in two years (2014 and 2015).

	Phenotype	All samples	2014		2015	
			Dyngby	Holeby	Dyngby	Holeby
Fraction mass	f2.8	82.2 ± 9.1	87.5 ± 6.2	87.6 ± 4.9	77.8 ± 9.0	78.8 ± 9.3
	f2.5	14.4 ± 7.4	10.0 ± 4.8	9.8 ± 4.3	17.6 ± 6.8	17.7 ± 7.9
	f2.2	2.5 ± 1.7	1.9 ± 1.3	1.5± 0.6	3.7 ± 12.0	2.6 ± 1.3
NIT measurements	Protein	9.2 ± 0.9	10.3 ± 0.5	9.4 ± 0.5	9.2 ± 0.6	8.3 ±0.4
	PY	8.3 ± 1.0	9.6 ± 0.6	8.3 ± 0.4	8.4 ± 0.7	7.4 ± 0.4
	TW	68.3 ± 2.0	66.8 ± 1.7	68.4 ± 2.9	68.5 ± 1.4	68.9 ± 1.4
	Ergosterol	13.2 ± 3.0	13.8 ± 2.7	9.0 ± 2.3	14.6 ± 2.1	14.0 ± 1.9

f2.8 = weight (g) of seed size fraction >2.8 mm, f2.5 = weight (g) of seed size fraction > 2.5mm & < 2.8mm, f2.2 = weight (g) of seed size fraction >2.2 mm < 2.5mm, Protein = protein content (TS %), PY = protein yield (hkg · ha^-1^), TW = test weight (kg · hl^-1^), Ergosterol = ergosterol content (%)

**Fig 3:**
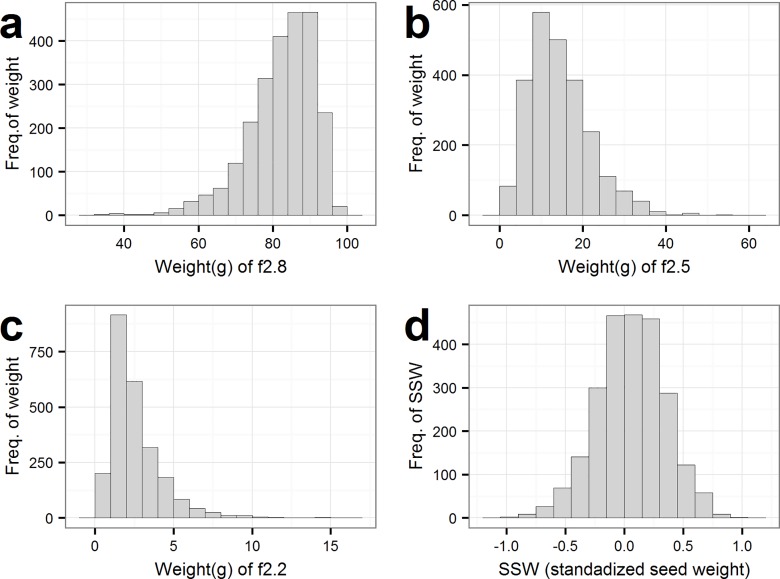
Distribution of seed size-parameters among the 309 barley lines. **a:** Weight(g) of f2.8, **b:** Weight(g) of f2.5 **c**: Weight(g) of f2.2 **d:** standardized seed size of f2.5 and f2.2.

The seed size fractions represent the weight of a given fraction, and as seen the largest fraction (f2.8) represents the main part of the seeds with a mean weight of 82.2 g. The largest coefficient of variation (CV) was found in the smallest fraction (f2.2) with a CV on 68% (results not shown). In addition, all seed size fraction weights were highly correlated to each other (results not shown). An overview of the seed size fraction weights in two years (2014 and 2015) and in the two locations (Dyngby and Holeby) is given in Table 2. In 2014, there was more variation in Dyngby compared to Holeby. In 2015, there was no major difference in the variation between the two locations. Furthermore, it is recognized that the mean of the largest fraction (f2.8) in 2014 are larger than in 2015. Fig 3 shows the distribution of each fraction size. The distribution of the largest fraction (f2.8) is positively skewed, while the two smaller fractions (f2.5 and f2.2) are negatively skewed. Therefore, the normally distributed SSW measure was created.

**Fig 4:**
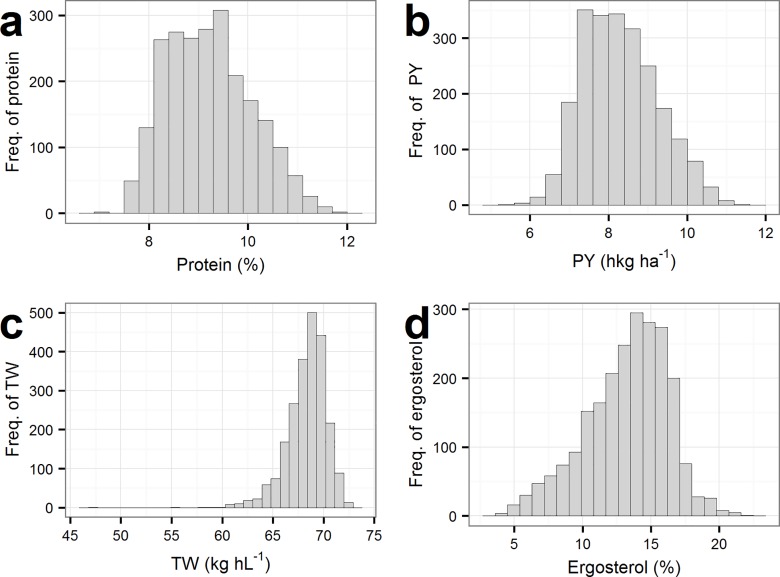
Distribution of NIT-parameters among the 309 barley lines. **a:** Protein (TS%) **b:** PY = Protein yield (hkg · ha^-1^), **c:** TW = Test weight (kg · hl^-1^) **d:** Ergosterol content (%)

An overview of the phenotypes i.e. protein content, PY, TW and ergosterol is given in Fig 4 and Table 2. The differences in the traits between the years and locations are seen from Table 2. Protein content was highest in Dyngby, both in 2014 and 2015. Samples with the highest protein content were found in 2014, and the variation was highest in 2015. The same tendencies were seen for PY. In general, little variation was seen in TW, but the most variation was found in Holeby in year 2014. In 2014, the difference between the locations was larger than in 2015, where almost no difference was seen. Ergosterol content was highest in Dyngby in both years. Ergosterol, protein and PY showed a normal distribution, while TW were slightly negatively skewed (Fig 4A–4D).

### Genomic prediction

Heritability and variance component estimates are seen from Table 3. As seen the variance of the error was highest for protein content and protein yield, which is further reflected by a lower heritability compared to the other traits.

**Table 3:** Heritability, maximum prediction accuracy and variance component estimates.

Phenotype	h^2^	Max acc.	Variance estimates			
			g	l	c	e
SSW	0.51 ± 0.06	0.72	0.14·10^−1^	0.09·10^−1^	0.06·10^−1^	0.09·10^−1^
Protein	0.21 ± 0.03	0.46	0.18·10^−1^	0.18·10^−1^	0.55·10^−1^	1.45·10^−1^
PY	0.26 ± 0.02	0.51	0.18·10^−1^	na	0.67·10^−1^	2.18·10^−1^
TW	0.51 ± 0.04	0.72	0.52	0.13	0.30	1.30
Ergosterol	0.61 ± 0.06	0.79	1.16	0.50	0.39	0.39

SSW = standardized seed weight, Protein = protein content (TS %), PY = protein yield (hkg · ha^-1^), TW = test weight (kg · hl^-1^), Ergosterol = ergosterol content (%)

The potential to use genomic prediction of quality traits in barley was investigated. First, the ability to predict quality traits of a variety when close relatives are in the training population was investigated. For this purpose, the cross-validation strategy was LOO. Accuracies were calculated for all traits as the correlation between the 309 GEBVs and the corrected phenotypes averaged over replicates for each line (Table 4).

**Table 4:** Prediction accuracies (acc.) using a leave-one-out (LOO) and a leave-family-out (LFO) cross-validation strategy. LFO was based on families including more than three lines, resulting in 45 families in total. Standard deviations are the standard deviation of the correlation coefficient.

Phenotype	LOO		LFO	
	Acc.	Bias	Acc.	Bias
SSW	0.68 ± 0.04	0.98 ± 0.06	0.63 ± 0.03	0.98 ± 0.05
Protein	0.40 ± 0.05	0.96 ± 0.12	0.26 ± 0.04	0.68 ± 0.10
PY	0.46 ± 0.05	1.04 ± 0.11	0.33 ± 0.04	0.87 ± 0.10
TW	0.63 ± 0.04	0.98 ± 0.07	0.56 ± 0.03	0.92 ± 0.06
Ergosterol	0.83 ± 0.03	1.02 ± 0.04	0.79 ± 0.03	1.02 ± 0.03

SSW = standardized seed weight, Protein = protein content (TS %), PY = protein yield (hkg · ha^-1^), TW = test weight (kg · hl^-1^), Ergosterol = ergosterol content (%)

The accuracies ranged from 0.40 for protein to 0.83 for ergosterol. To evaluate the level of the accuracy, the accuracies were compared to the maximum prediction accuracy being the square root of the line mean heritability. For all traits, the accuracy was close to the maximum possible prediction accuracy which indicates very high accuracies of the GEBVs.

### Prediction accuracies across progeny sets and families

After testing the general ability to predict the performance in the population (LOO in Table 4), the ability to predict the performance of a new set before the growth season was investigated by using a LSO cross-validation strategy. The prediction accuracies from the LSO are reported in Table 5.

**Table 5:** Prediction accuracies (acc.) using a leave-set-out (LSO) cross-validation strategy. LSO predictions are compared with leave-random-set-out (LSO_random_), using sets of same sizes as the progeny sets (S_2014_ and S_2015_). Standard deviations are the standard deviation of the correlation coefficient.

	Phenotype	LSO		LSO_random_	
		Acc.	Bias	Acc.	Bias
S_2015_ as training population	SSW	0.50 ± 0.08	0.57 ± 0.09	0.60± 0.07	0.96 ± 0.17
	Protein	0.22 ± 0.09	0.82 ± 0.3	0.22 ± 0.07	0.96 ± 0.24
	PY	0.19± 0.09	0.69 ± 0.3	0.22 ± 0.05	1.08 ± 0.20
	TW	0.49 ± 0.08	0.90 ± 0.15	0.40 ± 0.05	0.95 ± 0.06
	Ergosterol	0.79 ± 0.06	1.29 ± 0.06	0.76 ± 0.04	1.06 ± 0.13
S_2014_ as training population	SSW	0.52 ± 0.06	1.27± 0.15	0.63 ± 0.05	1.01 ± 0.17
	Protein	0.31 ± 0.07	0.87 ± 0.19	0.20 ± 0.02	0.94 ± 0.21
	PY	0.22± 0.07	0.71± 0.23	0.19 ± 0.02	0.98 ± 0.23
	TW	0.50 ± 0.06	0.94 ± 0.12	0.34± 0.03	0.94 ± 0.08
	Ergosterol	0.72 ± 0.06	1.29 ± 0.09	0.77 ± 0.04	1.14 ± 0.15

SSW = standardized seed weight, Protein = protein content (TS %), PY = protein yield (hkg · ha^-1^), TW = test weight (kg · hl^-1^), Ergosterol = ergosterol content (%)

Furthermore, for comparison the accuracy was calculated using randomly chosen sets (R_1_ and R_2_) being the same size as the progeny sets (S_2014_ and S_2015_) (Table 5). Using S_2014_ as a training population to predict S_2015_ gave the highest accuracies in all traits, except ergosterol content. With some phenotypes, the accuracy dropped when using the LSO, compared to the LSO_random_. However, the prediction accuracies were generally similar between the LSO and LSO_random_, although across set predictions were somewhat biased.

Secondly, the ability to predict across less related families was investigated by using a LFO cross-validation strategy. The prediction accuracy from most distant relative is seen from Table 4. Prediction accuracies were higher than the ones yielded by LSO in Table 5, while the accuracy of the LFO was lower than the LOO predictions. However, predictions of protein and protein yield were biased (Table 4).

### Effect of training population size and number of markers on prediction accuracy

**Fig 5:**
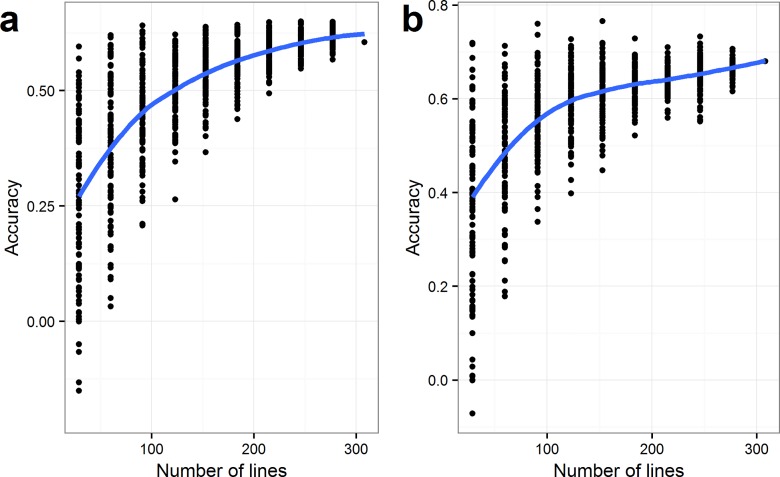
**Prediction accuracies with reduced training population size a:** Test Weight (TW) (kg ·hL^-1^) and **b:** SSW (standardized seed weight). Predictions were done for randomly selected line sets. The analysis was repeated 100 times for each step using a leave-one-out strategy. Curves were fit by loess.

To investigate the effect of the size of the training population, a similar experiment was performed using a leave-one-out cross-validation strategy. Thus, predictions were done using reduced training sets. The outcome for both TW and SSW is seen in Fig 5A and 5B. The two graphs show the same tendency; by reducing the number of lines in the training population, the mean accuracy of the replicated cross-validations decreased. The variation between the replicated cross-validations is also increased dramatically when using less than 200 lines. Using 217 lines for prediction of TW caused accuracies ranging from 0.49 to 0.66, while including only 62 lines in the training population resulted in accuracies ranges from 0.03 to 0.68.

**Fig 6:**
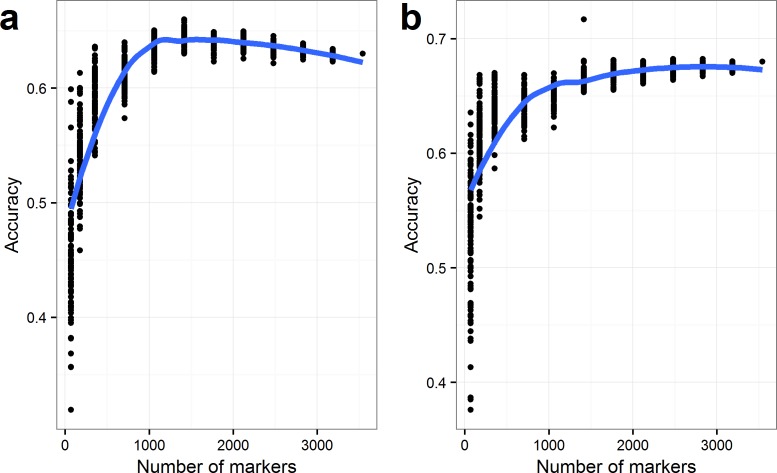
Prediction accuracies with reduced marker numbers. **a:** Test Weight (TW) (kg ·hL^-1^) and **b:** SSW (standardized seed weight). Predictions were done for randomly selected marker sets. The analysis was repeated 100 times for each step using a leave-one-out strategy. Curves were fit by loess.

The effects of the marker number included in the prediction were evaluated by reducing marker set used for prediction (Fig 6). The mean accuracy for both TW and SSW decreased with the reduction of the number of markers. In both cases there was a clear tendency in that the variation between the replicates increased using less than 1,000 markers, resulting in low prediction accuracies for some of the replicates. Additionally, the accuracy with some marker sets for SSW dropped already by using less than using 2,000 markers.

## Discussion

Genomic selection can be a valuable tool to accelerate genetic gains in plant breeding programs, and has been described as a valuable tool in several plant species (for example wheat and maize [12,34]). Genomic selection has only just started in barley breeding [35]. In this study, we explored the possibility of using genomic prediction in a commercial barley breeding program in northern Europe using seed quality traits as an example. The population consisted of 309 commercial lines, which may be a low number of lines compared to other species. The authors of [36] presented a genomic prediction study using 504 DH maize lines. However, it has been stressed that relatedness of training population and the test population also contribute to the prediction ability [16,18]. Since the linkage disequilibrium and family structure, which differ between species, influence prediction accuracy [37], the minimum size of the training population depend on the crop. A recent study showed that a training set of 100 elite barley lines is enough to obtain prediction abilities up to 0.8, depending on the trait [25]. This may partly be explained by the narrow genetic material in the elite barley lines used in the study of [25]. A study confirmed a low diversity in European barley compared to other continents [38]. Our results indicated that there was a strong genomic relationship between most of the lines in the population under study, making it suitable for applying genomic prediction models.

### Prediction accuracies and standard error estimates

Prediction ability is affected by several factors such as training population size, relatedness of training population with validation population and the genetic architecture of the traits studied. In this study, we found heritabilities of line means ranging from 0.21 for protein content up to 0.61 for ergosterol content. The results confirm that the most heritable traits display the highest prediction accuracies. The study of [25] detected a predictive ability of 0.521 for protein content using a training population of 424 lines. Our results showed a prediction accuracy of 0.40 for protein content with a training population size of 308. The heritability of protein content found by [25] was higher (0.66) than the heritability detected for protein in our study (0.21). The number of markers associated with the trait can influence the prediction ability [14]. Since, the lines used in [25] were elite lines already registered or candidates for registering, the higher accuracy can probably not be explained by fixation of QTL or allele frequencies. However, the lower prediction accuracy found in our study may be attributed to a lower heritability. Additionally, a reason for the higher heritability found by [25], may the different experimental setup which contain five locations and four years. Therefore, the expectation of prediction accuracy will also be higher, mainly due to more replications per line. As indicated by the variance components estimates (Table 3), the error estimates are high for some traits.

### Prediction ability across breeding sets and families

Genetic gains in breeding are obtained by selection of lines which are performing well in all environments in the coming years. Thus, the ability to predict the performance of lines to be tested next year is important. Therefore, we investigated the possibility of using S_2014_ lines to predict S_2015_ and vice versa. In general, the predictions were similar between the LSO and the LSO_random_. Compared to the LOO method, the accuracies decreased. Since the PCA plot indicated (Fig 1) that no strong genetic grouping based on years, the decreased accuracy was due to a decreased training set size. This was further confirmed by the comparison in Table 5, where prediction accuracies using random groups of the same size as the year groups, were similar. The predictions were in some cases biased for the prediction across the breeding sets (S_2014_ and S_2015_) which may indicate that the traits are influenced by genotype by environment interactions.

Lines in a breeding program are not necessarily closely related, and the ability to predict performance across less related families can be relevant in practical breeding. Therefore, the LFO cross-validation strategy was performed. The prediction accuracy of the LFO prediction were slightly higher than prediction across years, and lower than the LOO strategy accuracies. The training population was not extremely decreased when one family was left out compared to the leave breeding set out strategy, and the decrease in the accuracy was caused by a reduction in the genetic relationship between lines. Likewise, the low accuracy when predicting across breeding sets may be explained by a poorer family relationship across breeding sets. Several studies have noted that the accuracy of genomic predictions is highly influenced by the population used to calibrate the model [39,40].

### Effect of training population size and marker set size

Prediction accuracy is affected by training population size [9,16,41]. Our results showed a general decrease in prediction accuracy, when the size of the training population was decreased to below approximately 200 lines. The variation between the replicated predictions varied depending on family structure. Hence, high prediction accuracies were reached using a relatively small training population for some replicates. One explanation is that the investigation was based on variance components estimated from the full dataset. Estimating variance components on smaller data sets would cause lower prediction accuracies due to more imprecise estimates of variance components. Situations where variances are not well known would need larger training populations or the variance components should be estimated from historical material. Nevertheless, several authors stress the fact that small training population sizes may be enough if the relationship between lines is sufficiently described by the markers [25,39], indicating that the relatedness and allele similarities between and within the populations are as important as the number of lines. Nevertheless, for several reasons, in a commercial breeding program a number of factors need to be taken into account when defining the training population. Firstly, the optimal training population size may differ from trait to trait. Hence, in practical situations, the training population has to cover the need for all traits in the breeding program. Secondly, information about the lines may be limited in the first generations in a breeding program. However, in some cases variances may be obtained from historical material, so that variance components do not need to be estimated alone from recent material.

Results further showed that the prediction accuracy decreased, when the number of markers used for prediction was lowered. The decrease in accuracy could be explained by a reduction of markers in LD with the QTL. Hence, the number and distribution of markers must be enough to describe the relationships between lines. Results showed, that the prediction accuracy varied, not only due to the size of the marker set but depended on which markers were included. This highlights the fact that the number of traced QTL influence prediction accuracy, but also that the accuracy is influenced by the markers ability to capture family relationships between lines. Our results suggest that at least 1,000 markers are needed for accurate genomic predictions in barley. A minimum marker number of 1,000 markers may not be valid for all traits, since the two traits tested in this study show differences in their sensitivity to a decrease in marker density. Different traits may be more sensitive to the numbers of markers. When fewer markers were included low prediction accuracies were observed for some marker sets. If fewer markers are to be used, several factors should be considered. Firstly, the markers should be distributed all over the seven chromosomes. Secondly, the markers must be chosen for the specific trait, since the optimum marker set may not be the same for the different traits.

### Practical use in breeding

To our knowledge, no barley breeders in Northern Europe currently use genomic prediction routinely in their breeding programs. Our results showed high prediction accuracies for several traits using a training set of 308 lines. However, using less than 200 lines in the training set can result in low prediction accuracy, and the accuracy will be highly dependent on the genetic relationships between lines in training population and lines to be predicted. These results confirmed the results of [14,25], who indicate that the needed size of the training population is relatively small for commercial barley. In addition, our results indicated a minimum marker set of 1,000 to eliminate the risk of missing important parts of the genomic variance. For some traits, the prediction between breeding sets was low and biased. Optimum methods for genomic prediction of performance in unknown future environments need to be explored in future studies.

In conclusion, since the effective population size of elite barley lines is low, small training sets of around 200 lines is enough to obtain high prediction accuracies. However, the genetic relationship between the training population and the predicted genotypes is highly relevant. It is therefore recommended to use training sets of 200 lines or larger, if information about the training and validation sets is limited. The number of well-distributed markers is recommended to be at least 1,000 markers.

The prediction accuracies achieved using genomic selection were comparable to the maximum accuracies of predicting future performance. This corresponds to the accuracy of GEBVs that are close to unity. Very considerable gains can therefore be expected from the application of genomic selection in barley breeding since it would allow selection on large numbers of lines that only need genotyping and thus avoiding costly phenotyping for many traits. In addition, genomic selection can be applied earlier than phenotypic selection and thus reducing time from initial crosses to marketing. Implementation of genomic selectin in an early stage in a breeding program can increase the selection intensity. This is especially relevant concerning quality related traits, since these are often costly to measure. In general, our results confirm that high prediction accuracies can be obtained in barley breeding programs by implementing general methods for genomic selection. Therefore, genomic prediction has the potential to improve genetic gains in commercial barley breeding programs.

## Supporting Information

S1 Table Raw phenotypic data. (XLSX)

S2 Table Raw genotypic data. The 309 lines were genotyped by Illumina iSelect 9K barley chip. The genotyping was outsourced to TraitGenetics [27].
(CSV)
